# Young adults' healthcare utilisation and healthcare needs: Perceptions and experiences of healthcare providers

**DOI:** 10.1111/hex.13370

**Published:** 2021-10-08

**Authors:** Lisa Viktorsson, Eva Törnvall, Magnus Falk, Ingrid Wåhlin, Pia Yngman‐Uhlin

**Affiliations:** ^1^ Research and Development Unit in Region Östergötland, Department of Health, Medicine and Caring Sciences Linköping University Linköping Sweden; ^2^ Department of Health, Medicine and Caring Sciences, Management Department in Region Östergötland Linköping University Linköping Sweden; ^3^ Department of Health, Medicine and Caring Sciences, Primary Health Care Centre Kärna Linköping University Linköping Sweden; ^4^ Research Section Region Kalmar County Kalmar Sweden; ^5^ Department of Health and Caring Sciences Linnaeus University Kalmar Sweden

**Keywords:** content analysis, healthcare providers, healthcare utilisation, self‐care, young adults

## Abstract

**Background:**

Health care in many countries entails long waiting times. Avoidable healthcare visits by young adults have been identified as one probable cause.

**Objective:**

The aim of this study was to explore healthcare providers' experiences and opinions about young adults' healthcare utilisation in the first line of care.

**Method:**

This study used latent qualitative conventional content analysis with focus groups. Four healthcare units participated: two primary healthcare centres and two emergency departments. This study included 36 participants, with 4–7 participants in each group, and a total of 21 registered nurses and 15 doctors. All interviews followed an interview guide.

**Results:**

Data were divided into eight categories, which all contained the implicit theme of distribution of responsibility between the healthcare provider and the healthcare user. Young adult healthcare consumers were considered to be highly influenced by external resources, often greatly concerned with small/vague symptoms they had difficulty explaining and unable to wait with. The healthcare provider's role was much perceived as being part of a healthcare structure—a large organisation with multiple units—and having to meet different priorities while also considering ethical dilemmas, though feeling supported by experience.

**Conclusion:**

Healthcare personnel view young adults as transferring too much of the responsibility of staying healthy to the healthcare system. The results of this study show that the discussion of young adults unnecessarily seeking health care includes an underlying discussion of scarcity of resources.

**Patient or Public Contribution:**

The conduct of this study is based on interviews with young adult patients about their experiences of seeking healthcare.

## 1 INTRODUCTION

### 1.1 Background

Many (Western) countries struggle with long waiting times for patients to receive health care,^1^, ^2^ raising questions about healthcare utilisation and how much of it might be avoidable. The proportion of avoidable visits has been found to be approximately 12%–15%, but the definition of what is avoidable varies.^3^, ^4^ However, Parkinson et al.^5^ have defined three types of avoidable visits at emergency departments (EDs): divertible, preventable and unnecessary. Suggested solutions for handling avoidable visits are, for example, to refer inappropriate attenders away from EDs and secure faster access to outpatient services.^6^, ^7^ Though well intentioned, suggestions to prevent avoidable visits at EDs have been difficult to implement since only limited resources exist to maintain sufficient availability for minor illnesses at other facilities, such as primary healthcare centres (PHCs).^8^

Avoidable healthcare visits are well studied.^9^, ^10^, ^11^, ^12^ Part of that research indicates that a certain age group, young adults 20–29 years of age, accounts for a substantial proportion of the avoidable visits, all three types included, and that necessary actions should be aimed at them.^3^, ^13^, ^14^, ^15^ Research has shown that young adults in general prefer to seek health care over self‐care for minor illnesses.^16^ As newcomers to adult health care, obtaining health care without an adult advocate, young adults may lack the experience and knowledge needed to handle symptoms. Thus, health literacy could be a contributing factor. Health literacy is the capacity to access, understand, appraise and apply different types of health information to make decisions about health,^17^ and has been shown to be associated with healthcare utilisation.^18^

When studying avoidable healthcare visits, healthcare providers' perspectives and experiences of patients' healthcare utilisation are important factors to consider, since they influence patients' ability to achieve and maintain health and well‐being.^19^ The way in which patients (not least those with little experience of health care) are met by healthcare providers is likely to have an immense impact on how they will act in future situations of symptoms and healthcare seeking. For example, by using person‐centred attributes in healthcare meetings, such as being nonjudgemental, repeating information, encouraging questions and explaining with language appropriate to the patient, they increase health promotion and enable preventive practice.^20^

Avoidable healthcare visits in light of healthcare providers' opinions and experiences have been studied before,^7^, ^9^, ^21^ but less is known about providers' opinions and experiences in regard to young adults and their healthcare‐seeking behaviour in the first line of care, which is defined as the first possible entrance to healthcare services.

### 1.2 Aim of the study

The aim of this study was to explore healthcare providers' experiences and opinions about young adults' healthcare utilisation in the first line of care.

## 2 METHODS

This study used latent qualitative *conventional content analysis* ^22^ with focus groups for data collection. Focus groups were chosen since the research question was considered to require qualitative data in terms of discussions between informants with certain characteristics and knowledge about the topic. Also, the questions of interest formed an interview guide well designed for a focused discussion. Finally, during and after the focus group interviews, the researchers wanted to compare similarities and differences in and between different groups.^23^, ^24^

### 2.1 Setting

**Figure 1:**
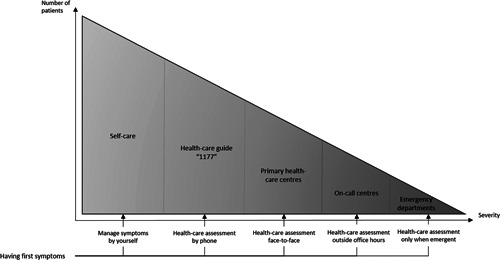
First line of care in Sweden

In Sweden, health care is publicly funded, with a healthcare organisation divided into 21 regions. This study took place in Southeast Sweden, including two regions with a total population of approximately 700,000 inhabitants. In Swedish health care, the system is structured for the majority of patients to seek health care at PHCs, preferably after contacting the Swedish healthcare guide service (named ‘1177’ after the telephone number to access the service) by telephone or online. The healthcare guide service guides the patient to either seek care, apply self‐care or wait. In urgent cases, the patient is expected to go to the ED. It is also possible to book an appointment at an on‐call centre after working hours (Figure 1). An initial screening and medical assessment is performed by registered nurses, both at EDs and PHCs. At EDs, this is done face to face and at PHCs, this is done mostly over the phone. If the initial medical assessment is considered urgent or needs a doctor's medical assessment, the patient is referred to a doctor immediately or booked for a doctor's appointment.

### 2.2 Study population and data collection

Data were collected using a convenient sample^25^ during the period of March to September 2019. To answer the research question representation from both PHCs and EDs, doctors and nurses and several regions were considered necessary. Therefore, four units were chosen, two PHCs and two EDs, allocated in two regions. For all units it was decided to have two focus groups each: One with doctors and one with registered nurses. Since focus groups should have not less than four participants, the research team concluded that at least 24 participants were required, which was considered enough for data collection. Both PHCs and EDs were chosen due to location and size. One unit was represented with only one focus group and one additional individual interview. On‐call centres were also represented in the interviews, since some of the informants were stationed at such units as well. Each focus group consisted of 4–7 participants, with a total of 36 participants in the study: 22 from PHCs and 14 from EDs, including 21 registered nurses and 15 doctors. The age of the informants ranged from 26 to 68 years, with a mean age of 39 years, with work experience ranging from 2 to 41 years (mean: 12 years). An invitation to participate together with information about the study was mediated through the unit operations manager to all coworkers meeting the inclusion criteria, that is, registered nurses and doctors working at the included units. Those wanting to participate reported their interest to the operation manager, thereby providing their informed consent to participate, and then the time and the place for the interviews were decided. Interviews were carried out at the providers' respective workplaces. All focus groups were held by two interviewers from the research team in different combinations. One acted as the moderator and the other interviewers acted as assistants to the moderator, taking notes and helping with further questions if needed. Before the interviews started, participants were informed about the study.

The interviews followed an interview guide and interviews started with an introduction, followed by an explanation of the aim of the study. All interviews then started with the following introductory question: *How do you experience young adults' health‐care seeking behaviour?* Followed by: *Would you please describe a recent meeting you had with a young adult? Do you think young adults seek the right level of care relative to their symptoms? What do you think about gatekeeping? Have you found that any care options are missing for young adults when they seek care? Do you have any thoughts on how the organisation could or should change?* Also, a conceptual model describing how young adults perceive seeking healthcare in the first line of care (yet unpublished study) was presented and discussed. In‐depth questions were asked when needed. Each interview lasted about 1 h, and  recorded and transcribed into full text. The project was conducted according to the ethical standards of the Helsinki Declaration. Ethical approval was sought from the Regional Ethical Review Board in Linköping, but was in their review considered not to be subjected to the Swedish Ethics Review Act, and thereby not in the need for ethical approval to perform. However, an advisory statement from the authority was given, in which no objections were raised (Dnr. 2018/129‐31).

### 2.3 Analysis

Data were analysed using conventional content analysis.^22^ All transcribed texts were read multiple times to gain an in‐depth understanding of the data. In a second step, data were analysed by coding; the text was read line by line and words or sentences of importance were highlighted. During the coding, the researcher took notes of ideas and impressions obtained from the text. After the initial coding, all codes were ordered into categories: subcategories and then main categories. This was done with the help of the notes. In this stage, all research interviewers participated in the analysis to verify that the chosen categories were consistent with the interviews. Some corrections were made, both of categories and of the grouping of codes. Finally, the relationships between categories were identified, creating an overarching theme.

## 3 RESULTS

**Figure 2:**
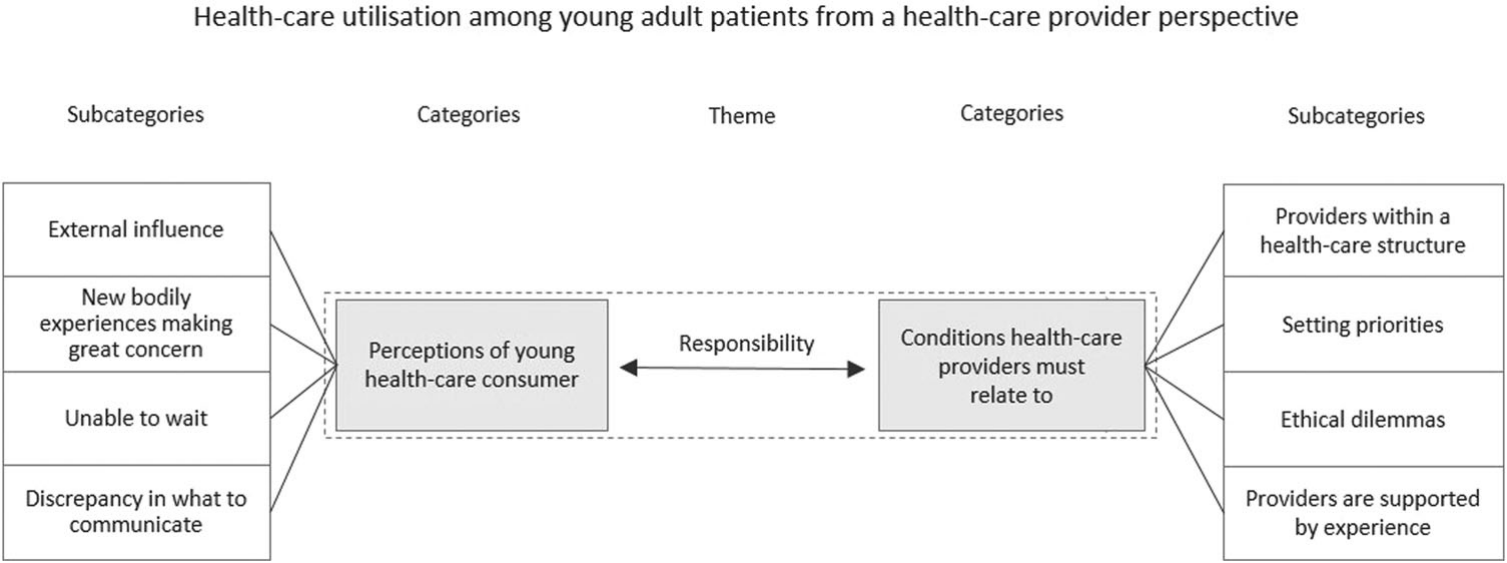
Healthcare utilisation among young adult patients from a healthcare provider perspective. Different underlying factors contribute towards taking or transferring responsibility. The dashed line denotes the healthcare meeting

Throughout the interviews, there was an underlying and unexpressed theme regarding the distribution of responsibilities between healthcare users and healthcare providers (Figure 2). Eight categories were coded from the data, all containing discussions of the distribution of responsibility depending on the situation. Healthcare providers perceived young adults as putting much of the responsibility of getting healthy on the healthcare system and unable to manage symptoms by themselves, but informants also discussed placing more responsibility on patients, as the system is unable to manage increasing healthcare needs. Healthcare providers presented an understanding of young patients' perceived needs, but also argued for actions needed from the healthcare system to prevent some of the healthcare‐seeking behaviour (number in brackets represent the different interviews).

…you come to the end of the road somewhere and the patient says ‘Yes, but I can't live with this’ but we don't see it as a serious illness or even something we can treat – more like they are handing the problem over to us. (3)

### 3.1 Perceptions of young healthcare consumers

#### 3.1.1 External influence

Healthcare providers recognized young adults as impressionable and influenced by their parents, media and other sources of information, and especially by what is referred to in this study as ‘Googling’. Informants described the impact of media on patient meetings in both positive and negative terms, reflected for example in no longer having to argue about restricting antibiotics prescription to the same extent as before, but in contrast, as another example, needing to defend why they do not immediately refer for an magnetic resonance imaging for an aching knee.

I know that there have been some occasions when people have gone straight to reception [Accident & Emergency Department] and said “Can I have an MRI scan?” because they have Googled it and decided that's what they need… …But I think the MRI idea comes from the sporting world with all the elite athletes who have suddenly had a scan in the middle of the night on their knee, for example… (8)

Googling was described as common in all ages, though the application of information was perceived to differ between young adults and older age groups. Informants, mostly doctors, emphasized Googling as a good entry point to conversation and a helping hand in putting patients on the right track regarding their symptoms. Overall, young adults were perceived as well informed, but having difficulty in distinguishing between relevant and irrelevant information. Their seeking behaviours were perceived to demonstrate a lack of knowledge of when and where to seek health care and for what symptoms. Common in all focus groups was discussion about the need for information about health care and self‐care, preferably to be taught in schools.

I've thought about this before and I think it would be a good idea for people to have some general health education… similar to having home economics classes at school; you would have some medical lessons at school. (5)

Unlike older age groups, parents of young adults were perceived as contributing to young adults' healthcare‐seeking behaviour. Examples given were as follows: Young adults being advised to seek health care by their parents, parents calling the provider for their adult child and patients showing signs of having ‘helicopter parents’, parents overinvolved in their children's life.

#### 3.1.2 New bodily experiences raising great concern

Healthcare providers reported that young adults frequently sought care for symptoms that were difficult to diagnose. Symptoms like abdominal pain and chest pain, sometimes with underlying psychological causes, were described as common. These types of symptoms were often considered by informants as harmless and part of normal body experiences. Body experiences, symptoms that should not be, or in some cases could not be, treated. Nevertheless, informants expressed a need to take patients' worries seriously and highlighted the importance of trying to calm them and reduce their distress. Having the time to talk with the patient was presented as a factor affecting healthcare providers' ability to ease those worries.

A group that you perhaps don't take entirely seriously because there's so much worry in the group as a whole, or maybe regard as people who look for quite minor symptoms and they have numerous investigations without them resulting in a somatic diagnosis… often you just calm these kind of patients down and there's nothing more you can do. (7)

#### 3.1.3 Unable to wait

Self‐care was not perceived as something commonly performed by young adults. Healthcare providers encountered few patients in this age group who had read about self‐care advice or called the healthcare guide service before seeking health care. Young adults were perceived as seeking care for symptoms that were often vague and of short duration, and without having tried painkillers when appropriate. The perception was that young adults demand immediate help and to instantly get healthy, implicitly asking for a quick fix.

I think that younger people have different ways of seeking help in comparison with the older generation in that they want help with more trivial things and are less patient about waiting for things. (1)

The need for guarantees was pointed out as important to young adults. A never‐ending demand for inspections, examinations and confirmations that all is well was described. Young adults, to a greater extent than other age groups, were perceived to believe that they are entitled to every test, examination and medicine available. Informants perceived young adults as reluctant to seek help from anyone other than registered nurses and doctors, considering parents', other relatives' and even pharmacists' knowledge and experiences insufficient. Young adults were perceived as wanting guarantees and assessments from a healthcare professional.

And people are not really able to think for themselves either, I feel. Something like: is it reasonable that I should request hospital transport because I have broken my hand and went to Accident and Emergency department on a Thursday night, is that actually reasonable?… Because I find that the aspect of whether something is reasonable or not is often missing… (8)

#### 3.1.4 Discrepancy in what to communicate

Communicating with young adults was not considered to be a problem. However, obtaining good, satisfactory medical history was considered to be difficult at times, especially from younger men. Young adults were perceived as novice patients who have difficulty providing concrete medical history, and sometimes, informants perceived patients to be exaggerating their symptoms. There were also examples of headstrong, belligerent and even arrogant patients threatening to report or frame healthcare providers.

Informants described many different ways to converse with young adults. Doctors described trying to educate patients while treating them and also working with person‐centred care. Registered nurses described how they sometimes try to go the extra mile when needed, by explaining the diagnosis, confirming that the patient understands it and doing a final check that the patient is satisfied with the information provided. Both doctors and registered nurses emphasized the difficulty of making that little extra effort, given the demand to prioritize patients with more serious illnesses and the lack of time. However, treating young adults with professionalism and providing clear explanations were highlighted as important considerations when meeting patients in this age group.

But if symptoms are quite unclear, the onus is more on the patient to describe and explain them. And in this age group, I don't know if it's unique to them, but it was often extremely difficult to communicate their symptoms. They don't really have such a good understanding, for example, of taking a medical history. They don't understand why these questions are relevant. (3)

### 3.2 Conditions that healthcare providers must relate to

#### 3.2.1 Providers within a healthcare structure

Being part of a healthcare structure was one recurring topic. The first line of care consists of many units and activities, and a focus of discussion was often whether or not patients were at the right place at the right time. Healthcare providers described young adults frequently seeking care with symptoms that could or should have been managed elsewhere. Most common were patients without urgent symptoms seeking care at EDs or on‐call centres when they should have been managed at PHCs over the next few days.

All possible entries to health care were described as separated from each other, with the healthcare guide service perceived almost as a lonely island. The healthcare guide service was considered to be generous with referrals, and also recognized for having a difficult task in trying to determine symptoms only by phone.

No, and it's a big responsibility for us when we are sitting there er… we are expected to be good at most things, but it is emergency care that we are good at. We're not a health centre and we're not so good at health centre cases. That's the way it is – it's really difficult. (2)

Informants reported that there were too many possible entries to health care and also that the continuity of care was perceived to have worsened. On‐call centres were given as an example of enabling unnecessary care, with young adults considered over‐represented because the centres allow self‐referral. Simultaneously, informants emphasized the possible need for a separate entry to care for mental health and availability of a support person. Informants discussed the contradiction of a patient having to first meet with a doctor before referral to a psychologist, which increases the risk of high rates of prescription of antidepressants due to limited knowledge.

#### 3.2.2 Setting priorities

In the first line of care, triaging/gatekeeping was described as an obvious part of everyday work for registered nurses, at both EDs and PHCs. In addition, doctors at PHCs emphasized performing gatekeeping when considering whether to refer a patient to specialist care. Registered nurses were, overall, confident in their assessments. They noted facing resistance from some patients, but that once they clarified the necessary priorities, most patients accepted them. Triage over the phone was sometimes perceived as complicating an assessment, because of not being able to see the patient—which is the case at PHCs—but overall, registered nurses were assertive when triaging. Among doctors, the discussion of triage focused mainly on giving compliments to the registered nurses about their ability to triage. They considered triaging/gatekeeping as necessary, but were hesitant to discuss whether a visit could be considered unnecessary or not, as only the visit itself allowed the determination of whether the visit was necessary or unnecessary. An additional point made in the discussion was that the patient has already done a first triage of his or her own before even contacting the healthcare system.

An emergency was defined and used differently depending on the healthcare unit, healthcare provider and situation. Healthcare providers at EDs viewed young adults as misunderstanding what should be considered an emergency and ignorant of which assessments/treatments were being performed at EDs and which were not. Also, young adults were considered to define injuries and illness as more emergent than healthcare providers. At EDs, the severity of illness was highlighted as crucial, before being pleasant.

Because when you also say ‘your symptoms aren't acute’ and explain ‘that we look after emergencies here, that for example a heart attack is an emergency’ and then you get an answer like ‘But I'm seriously ill’, they don't really understand the difference. (2)

At PHCs, registered nurses often discussed the degree of emergency when describing young adults' healthcare‐seeking behaviour. Registered nurses described having what are called ‘emergency appointments’ available when booking a patient for a doctor visit. For one of the two PHCs, this was the only way for registered nurses to book patients without having to consult with a doctor. These appointments are few and need to be restricted to patients with urgent symptoms. Accordingly, discussions of what should be considered emergent highly apparent not only at the EDs but also at PHCs.

‘I feel feverish’, ‘Yeah, but have you’ ‘No, I haven't got a thermometer’ and then, OK, you just have to say something like ‘Start by trying to find a thermometer so that you can take your temperature’… …I understand that people can feel really ill, but the feeling of being very ill isn't something that… that makes a doctor's assessment a priority. We prioritise based on… other medical criteria [Assessment by phone]. (6)

#### 3.2.3 Ethical dilemmas

Several ethical dilemmas were discussed within all focus groups based on the perception that young adults sometimes seek care for trivialities. Informants raised the question of what health care *should* do in contrast to what health care *could* do on numerous occasions. Also raised were the questions of what is considered medically defensible and what is reasonable to request of publicly funded health care.

So it's an ethical issue that probably wasn't a problem until more recent times. On the one hand, an awful lot more could be done all the time and more and more things can end up in the frame for what is treatable. Because it's not just about…, what we do, but about how much we should do? Like where's the limit? (5)

Informants pointed out a feeling of contradiction, being unable to treat every patient they were forced to triage and at the same time having to deal with requirements for increased accessibility from top executives. They described experiencing frustration meeting young adults unaware of the rules and with laws that healthcare providers have to relate to and follow. Laws were sometimes perceived as contradicting each other: For example, a statutory guarantee of receiving healthcare within a defined number of few days versus conflicting regulations aiming to put those with the greatest needs first.

#### 3.2.4 Providers are supported by experience

Experience played an important role in how healthcare providers chose to respond to a patient's request. Past experience weighed heavily when performing assessments. With experience came comfort, with sometimes having to make decisions without complete information. Experience was also reflected in discussions, when providers pointed out that some aspects of what was being discussed were not unique to young adults.

Healthcare providers pointed out that young adults rarely suffer from anything serious. At EDs, experiences involving young adults who showed no evident signs of why they sought health care were described. Doctors, both at EDs and PHCs, described this age group as sometimes seeking care for trivialities, but for this reason, this type of visit was also rewarding, as treatment was easy and therefore not seen as a problem. With experience also came generalisation and stereotypes, which were discussed openly in some focus groups, while only implied in others. Informants described young adults as a statistically healthy group. In discussion, this emerged when doctors explained sometimes being restricted with further actions like taking samples and referring to a specialist, justifying this by stating that sometimes waiting is a better approach.

And so 20‐29 year‐olds are generally very healthy. They rarely have any serious illnesses, and if they do, it's usually something they have had all their lives. It is very rare that they suffer from any…. dire emergency… (8)

## 4 DISCUSSION

Young adults' healthcare utilisation, seen from a healthcare provider perspective, is a question of distribution of responsibility. Providers' experiences of young adults as easily influenced, with great concerns and infrequent use of self‐care, together with a healthcare structure that forces providers to conform to rigorous priorities, were manifested in the results. Implicitly, healthcare providers perceive young adults as an age group that has difficulty interpreting and evaluating symptoms, and is reluctant to wait and thereby take responsibility for managing symptoms by themselves.

To understand the perceived distribution of responsibility in a healthcare setting, there needs to be a clarification of the governance behind that healthcare setting. Sweden is defined by Espling‐Andersen^26^ as a social democratic regime, a welfare state taking a high degree of responsibility for its citizens by guaranteeing basic services for them. In return, the regime secures functionality for the state itself by having functional citizens.^26^, ^27^ Earlier research exploring an egalitarian state healthcare system, comparable to Swedish health care, has argued that healthcare utilisation can be viewed as a moral process, with citizens trying to balance their rights with their responsibilities. This is done by contributing to society and requesting only what is needed. Translated to a healthcare context, this means trying to distinguish normal bodily changes from sensations that are actually symptoms of illness needing health care.^28^ The results of this study show that healthcare providers perceive young adults as having difficulty determining this difference. This is supported by Gustafsson et al.'s^16^ finding that young adults are insecure in handling symptoms by themselves.

Ahola‐Launonen^27^ has discussed trends of ‘responsibilising the individual’ and the need for reciprocation. With today's trend towards individualism, there is a risk of neglecting the need for acting with reciprocity in the bigger picture.^27^ Young adults may demand health care that is perceived as unnecessary, though receiving more supporting health care in early life could yield more self‐care later on. Further, Ahola‐Launonen^27^ argues that discussions of rights and responsibilities should never be based on scarcity of resources. By supplying patients' healthcare needs in the first place, we might prevent much higher costs to society later on. In this study, healthcare providers identified young adults as a rewarding group to work with, often having minor and easy‐to‐handle symptoms. The opportunity to help ease worries and anxiety when time allowed was also mentioned as worthwhile. For healthcare providers, having those extra 5 min for handling young adults' worries about symptoms could facilitate young adults' ability to manage self‐care the next time symptoms occur.

The first line of care is supposed to care for sicknesses and injuries, both emergent and nonemergent, at EDs and PHCs, respectively. Nevertheless, what can be considered acute or not is a frequent topic among healthcare providers at PHCs as well as EDs. This could be partly explained by PHCs frequently using what they call ‘acute appointments’, which are the appointments available for registered nurses when booking patients within the next 24–48 h. The appointment booking procedure looks somewhat different depending on the region, but, overall, the word acute is frequently used. For some of the registered nurses, booking acute appointments is the only way for them to book patients without having to check with a doctor first. This can be time consuming; consequently, the assessment of whether or not a patient is having an acute problem can be considered highly relevant. Seeking out‐of‐hours care for minor illness has been thought of as a problem for a long time in health care.^29^, ^30^ A Norwegian study has shown that young adults are a large group among patients seeking out‐of‐hours care, and explained that this age group may have a lower threshold for seeking help.^31^ On the other hand, a Danish study has shown that out‐of‐hours patients are not trying to see the doctor ahead of those with more severe symptoms.^32^ Perhaps the fact that young adults seek care out‐of‐hours is just a sign that people need health care to be available outside of working hours. Also, given the difficulty of getting an appointment for nonacute symptoms, patients probably seek care where possible and doing so out‐of‐hours enables self‐referral. This could be a sign that patient flows have changed due to healthcare system errors rather than an explanation of why patients seek unnecessary health care.

Young adults are perceived as an age group with many worries and much anxiety about symptoms. A systematic review examining healthcare‐seeking behaviour in cancer and acute myocardial infarction found that people seek care differently, depending on the type of fear they have about their symptoms. The authors examined five stages of fear: ‘being worried’, ‘having fear’, ‘being anxious’, ‘being in panic’ and ‘feeling death anxiety’. They found that individuals who experienced anxiety, panic and death anxiety sought health care sooner than those who experienced being worried or had fear. Worrying and having fear could, on the contrary, cause delayed healthcare seeking.^33^ Healthcare providers show a willingness to help young adults with worries and anxiety about their symptoms, though the discussion about being able to help was repeatedly affected by the fact that there are limited resources and a consequent need to perform gatekeeping, also part of the discussions of ethical dilemmas. Gatekeeping is the one factor separating the experiences of registered nurses from those of doctors. While doctors might find it somewhat irrelevant to discuss unnecessary healthcare seeking, as it is only determinable as such after the fact, registered nurses tend to consider it to be more important. Registered nurses have to quickly first assess whether or not the patient needs to see a doctor, either by triaging (EDs) or by phone assessment (PHCs). An ethnographic field study at emergency primary care clinics in Norway has shown how gatekeeping can be quite a struggle for nurses.^34^ The study revealed three main concerns with gatekeeping and low‐priority patients: Sympathy for patients having to wait several hours, the negative impact on nurses' everyday work and concern about providing inconsistent service by giving low‐priority patients time during periods when there are few patients. The study discusses how an antagonistic tendency in patient–provider relations has to be recognized for its structural foundations and not be seen as a moral shortcoming of nurses. By extension, the research highlights how rights and responsibilities are at risk of being reduced to a discussion solely about resource scarcity.^27^

Earlier research has shown that health literacy and healthcare utilisation are linked.^18^ Health literacy is a recurring theme that emerged in the results of this study, mostly implicitly in terms of perceived ignorance. Healthcare providers acknowledge that young adults are not health experts and sometimes need help to sort out what they suffer from. However, healthcare services' limited resources affect healthcare providers' ability to fully accept young adults' perceived need for care. According to Parker  and Ratzan^35^ there is a dual nature of communication enabled by health literacy when the skills and abilities that a person possesses are aligned with the complexity and demands needed for health. Limited resources in health care could be a contributing factor to the inability to keep the demands and complexities of health information and health tasks aligned with young adults' skills and abilities, thereby preventing them from achieving optimal health. Without free access, people turn to other sources of information. Healthcare providers see young adults as being influenced by many different sources, the internet being one. As Tonsaker et al.^36^ discuss in their research, health care provider have to face that internet is a major source of patients' health knowledge. Healthcare providers show their understanding of young adults' use of the internet by sometimes opening their conversation by asking what the patient has googled, as a way to ensure that they are on the same path in decision‐making. As our results show, the informants found that young adults sometimes have difficulty explaining their symptoms. Asking about Googling may be one of many constructive ways to help the patient start a conversation about his or her symptoms. To return to the earlier discussion about responsibilities, healthcare providers' suggestion that basic body awareness be taught in school might increase young adults' sense of responsibility for their own health.

### 4.1 Strengths and limitations

The researchers involved in the interviews and analysis all have previous experience with qualitative studies. The moderator is a public health scientist with no relationship to any of the participants, which we hope facilitated a climate of talking freely. The interview assistants are all registered nurses and added questions if needed. The results from this study could be transferable nationwide since many of the conditions that healthcare providers have to relate to are the same in the whole country.

Using a conventional content analysis enabled to find the latent content in the data, presented here as the distribution of responsibility. The results highlighted both what was implied and what was said explicitly.^37^ The decision to use focus groups was based on Morgan's^24^ inclusive description of collecting data through group interaction on a topic determined by the researcher. Ethical dilemmas were one example of a topic where focus groups facilitated discussions by informants giving each other questions, thereby increasing the researchers' understanding. Occasionally, the discussions became intense and gave the research group a deeper understanding of how some questions engage healthcare providers at several levels.

The study has a number of limitations. Each group consisted only of members from the same respective healthcare unit. This may have helped to provide an open climate for discussion but may also have allowed relationships between coworkers to be part of the discussion. Also, putting together a focus group consisting of coworkers may stifle free expression that may adversely affect future everyday work relations. Coworkers were placed in the same group mainly because of difficulties in gathering a group at all. The interviews were made possible by the ability to arrange them during lunch, and this is also the main reason why the informants needed to be from the same unit. The research group considered the discussion climate to be open overall. There was some perceived restraint in one focus group due to an imbalance of hierarchy between doctors, but the interviews were still fruitful, and this group was probably offset by the relatively large number of other focus groups.

## 5 CONCLUSION

Healthcare personnel view young adults as transferring too much of the responsibility for staying healthy to the healthcare system. Though young adults are often considered to have symptoms that are easy to handle, those same symptoms cause a perceived battle of responsibilities because they repeatedly need to be deprioritized. The results of this study show that the discussion of young adults unnecessarily seeking health care includes an underlying discussion of scarcity of resources.

## CONFLICT OF INTERESTS

The authors declare that there are no conflict of interests.

## AUTHOR CONTRIBUTIONS

Lisa Viktorsson is the main author and contributed the most to the study in terms of data collection, data analysis and manuscript writing. She also acted as the moderator for all focus groups interviews. Pia Yngman‐Uhlin contributed towards designing the study, collecting data, analysing data, manuscript editing and as an interview assistant. Ingrid Wåhlin contributed with data collection, data analysis, manuscript editing and acted as a moderator for the individual interviews and as an interview assistant. Eva Törnvall contributed towards designing the study, analysing the data, manuscript editing and as an interview assistant. Magnus Falk contributed towards designing the study and manuscript editing.

## Data Availability

Data are available on request due to privacy/ethical restrictions.
